# Unveiling the intricate causal nexus between pancreatic cancer and peripheral metabolites through a comprehensive bidirectional two-sample Mendelian randomization analysis

**DOI:** 10.3389/fmolb.2023.1279157

**Published:** 2023-10-25

**Authors:** Rong Sun, Haoyu Xu, Feng Liu, Bin Zhou, Minli Li, Xiangdong Sun

**Affiliations:** Jinling Hospital, Affiliated Hospital of Medical School, Nanjing University, Nanjing, China

**Keywords:** pancreatic cancer, peripheral metabolites, two-sample Mendelian randomization, high-density lipoprotein, very low-density lipoprotein

## Abstract

**Aim:** Pancreatic cancer (PC) is a devastating malignancy characterized by its aggressive nature and poor prognosis. However, the relationship of PC with peripheral metabolites remains not fully investigated. The study aimed to explore the causal linkage between PC and peripheral metabolite profiles.

**Methods:** Employing publicly accessible genome-wide association studies (GWAS) data, we conducted a bidirectional two-sample Mendelian randomization (MR) analysis. The primary analysis employed the inverse-variance weighted (IVW) method. To address potential concerns about horizontal pleiotropy, we also employed supplementary methods such as maximum likelihood, weighted median, MR-Egger regression, and MR pleiotropy residual sum and outlier (MR-PRESSO).

**Results:** We ascertained 20 genetically determined peripheral metabolites with causal linkages to PC while high-density lipoprotein (HDL) and very low-density lipoprotein (VLDL) particles accounted for the vast majority. Specifically, HDL particles exhibited an elevated PC risk while VLDL particles displayed an opposing pattern. The converse MR analysis underscored a notable alteration in 17 peripheral metabolites due to PC, including branch chain amino acids and derivatives of glycerophospholipid. Cross-referencing the bidirectional MR results revealed a reciprocal causation of PC and X-02269 which might form a self-perpetuating loop in PC development. Additionally, 1-arachidonoylglycerophosphocholine indicated a reduced PC risk and an increase under PC influence, possibly serving as a negative feedback regulator.

**Conclusion:** Our findings suggest a complex interplay between pancreatic cancer and peripheral metabolites, with potential implications for understanding the etiology of pancreatic cancer and identifying novel early diagnosis and therapeutic targets. Moreover, X-02269 may hold a pivotal role in PC onset and progression.

## 1 Introduction

Pancreatic cancer (PC) is one of the most lethal forms of solid malignancy with a 5-year survival rate lower than 9%, which poses a formidable challenge to global health (Qin et al., 2020; Casolino et al., 2021). The annual incidence and mortality rates of PC are on a steady rise, showing minimal advancements in overall survival outcomes (Khalaf et al., 2021). The asymptomatic nature and rapid metastatic spread of PC contribute to the diagnosis at advanced stages, significantly limiting treatment options and leading to dismal survival rates (Huang et al., 2019; Chen et al., 2021). Another reason of PC’s unfavorable prognosis is that surgery stands as the sole curative approach. Thus, early diagnosis and novel treatment strategies for PC are eagerly wanted.

Metabolic reprogramming, recognized as an emerging hallmark of cancer (Hanahan and Weinberg, 2011), has garnered renewed attention over the last decades (Leal-Esteban and Fajas, 2020; Infantino et al., 2021). Metabolites, whether present within the local tumor or circulating in the peripheral blood, play a multifaceted role. They not only modulated energy dynamics but also orchestrated signal transduction among malignant and nonmalignant cells in tumor microenvironment (Garcia-Bermudez et al., 2018; Schworer et al., 2019; Pavlova et al., 2022). Beyond the local tumor site, the intricate reprogramming of tumor metabolism can exert far-reaching consequences on the host’s systemic metabolic landscape, and further leads to cachexia and antitumor immunity deficiency (Porporato, 2016; Drijvers et al., 2020; DePeaux and Delgoffe, 2021). Indeed, observational studies have reported associations between specific peripheral metabolites in PC patients, including branched-chain amino acids (BCAAs), glycerophospholipids, lipids and lipoprotein (Mayers et al., 2014; Shu et al., 2018; Stolzenberg-Solomon et al., 2020; Elebo et al., 2021; Encarnacion-Rosado and Kimmelman, 2021; Wang et al., 2022). Specifically, elevated plasma levels of BCAAs, including leucine, isoleucine and valine, are associated with an increased risk of future PC diagnosis (Mayers et al., 2014). The glycerophospholipids were preferred to be associated with decreased risk of PC (Shu et al., 2018). Lipids exhibited various effects on PC. Steroid hormones, such as androgenic steroids, pregnenolone steroids and corticosteroids, showed an inverse association with incident PC. Conversely, fatty acids and glycoursodeoxycholic acid sulfate displayed a positive association with PC (Wang et al., 2022). Regarding lipoproteins, various lipoprotein parameters, such as size, particle number, and lipid concentration (cholesterol and triglycerides) in the primary classes of lipoproteins, including very-low-density lipoprotein (VLDL), intermediate-density lipoprotein (IDL), low-density lipoprotein (LDL), and high-density lipoprotein (HDL), showed associations with PC. The size of HDL particles exhibited a positive correlation with PC stage, whereas HDL cholesterol levels and HDL particles showed opposite associations (Elebo et al., 2021). However, these findings originate from observational evidences, which are constrained by sample size limitations and beset by inherent biases, notably including reverse causation and confounding factors. Addressing this causal uncertainty of peripheral metabolites and PC is essential for understanding the potential therapeutic implications and identifying novel targets for interventions.

Recognized for its robustness, Mendelian randomization (MR) leverages genetic variants as instrumental variables (IVs) to unravel causal associations within observational datasets. In this study, we present a comprehensive bidirectional two-sample MR analysis to disentangle the reciprocal effects between peripheral metabolites and pancreatic cancer development. We identified 20 peripheral metabolites as potent contributors to PC while 17 were regulated by PC. Notably, our results revealed mutually positive causal relationship of X-02269 and PC, highlighting that they might form a vicious circle in PC onset and development. Through this investigation, we endeavor to unravel novel insights into the dynamic interplay between peripheral metabolites and PC, contributing to the broader understanding of PC etiology. The identified metabolites hold promise for the development of early diagnosis and targeted interventions for PC, thus mitigating the burden imposed by this formidable malignancy.

## 2 Materials and methods

### 2.1 Study design

**FIGURE 1:**
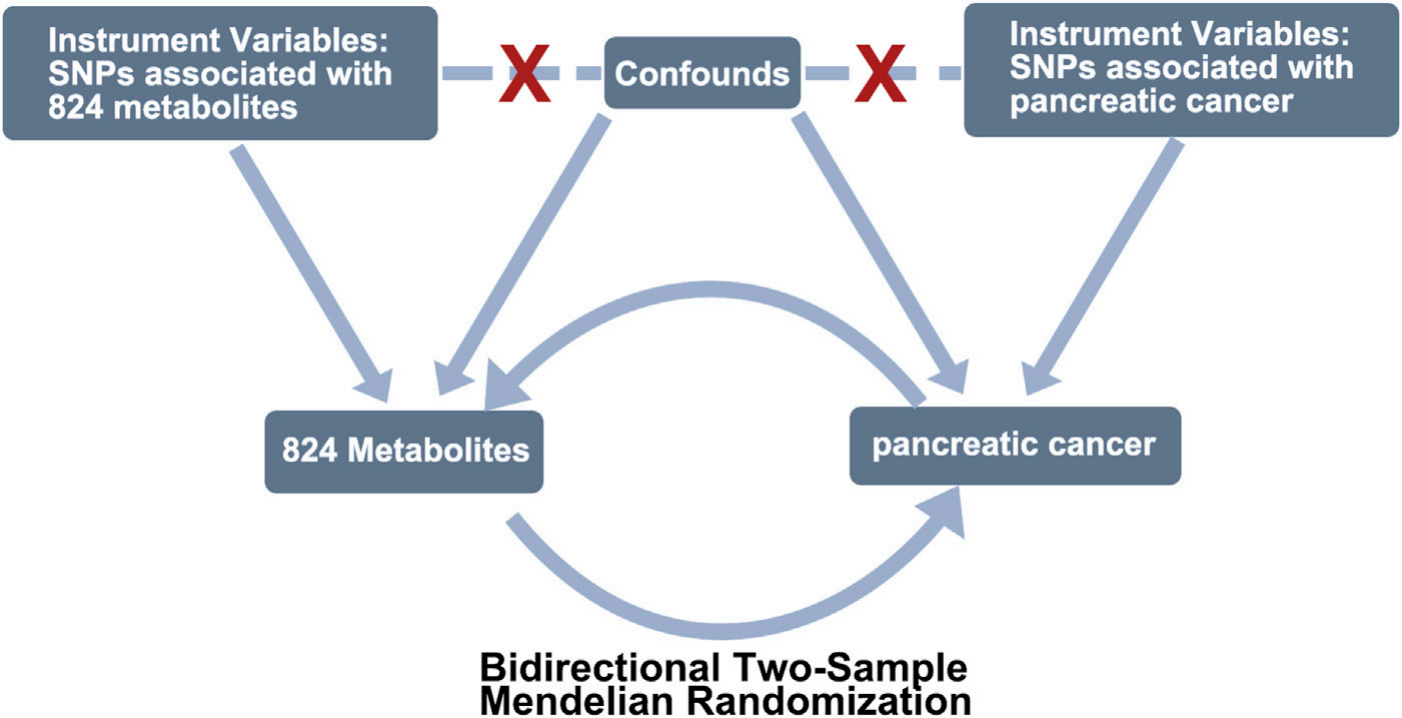
Study design of exploring the causal relationship between pancreatic cancer and peripheral metabolites by bidirectional two-sample Mendelian randomization. COPD, chronic obstructive pulmonary disease; SNPs, single-nucleotide polymorphisms.

We employed a bidirectional two-sample MR analysis (Figure 1) to investigate the causal relationship between PC and 842 peripheral metabolites in European-ancestry datasets. The genetic instruments utilized in our study met the three essential assumptions of MR analysis. Firstly, the selected genetic variations, serving as instrumental variables (IVs), exhibited significant associations with the exposure of interest. Secondly, these genetic variations were independent of both known and unknown confounding factors. Lastly, the genetic instruments were specifically associated with the outcome, establishing a plausible causal pathway.

### 2.2 Data source

Genetic associations pertaining to human peripheral metabolites were extracted from three distinct research studies (Shin et al., 2014; Kettunen et al., 2016; Borges et al., 2020), which were accessible through MRC Integrative Epidemiology Unit (IEU) OpenGWAS project (https://gwas.mrcieu.ac.uk/). Correspondingly, genetic associations concerning pancreatic cancer were acquired from the FinnGen database (R8 level), encompassing a sample size of 1249 cases and 259583 controls. It is noteworthy that all conducted GWAS involved participants of European ancestry. The comprehensive specifics regarding the sourced GWAS datasets are meticulously outlined in Table 1.

**TABLE 1:** The collected GWAS datasets of peripheral metabolites and pancreatic cancer.

Trait	Database	Label	Metabolites	Sample size
Peripheral Metabolites	MRC IEU OpenGWAS	met-a	452	7284
Peripheral Metabolites	MRC IEU OpenGWAS	met-c	249	115078
Peripheral Metabolites	MRC IEU OpenGWAS	met-d	123	24925
Pancreatic Cancer	FinnGen	FinnGen_C3_PANCREAS_EXALLC		260832

### 2.3 Mendelian randomization analysis

In preparation for the MR analysis, a meticulous selection process was meticulously undertaken to identify genetic IVs in adherence to specific criteria. Initially, the selection criterion mandated that single nucleotide polymorphisms (SNPs) demonstrate a statistically significant association with the exposure at the genome-wide significance threshold. Subsequently, an extensive range of tests spanning from 5 × 10^−8^ to 1 × 10^−6^ were meticulously performed to discern the optimal *p*-value threshold for peripheral metabolites and pancreatic cancer, respectively. The chosen thresholds, 5 × 10^−8^ for peripheral metabolites and 5 × 10^−6^ for pancreatic cancer, were primarily grounded in the abundance of informative SNPs. Furthermore, SNPs with a limited association strength with telomere length (F-statistic ≤10) were judiciously excluded from consideration. The F statistic was calculated employing the equation: $F={\left(\frac{beta}{se}\right)}^{2}$ . Finally, the chosen SNPs were subjected to stringent scrutiny for conditional independence and absence of linkage disequilibrium (r2 < 0.001), bolstered by a minimum clump distance exceeding 10,000 kb. These rigorous selection criteria were meticulously applied employing the European 1000 Genomes Project reference panel.

Upon the harmonization of exposure and outcome summary data, the central MR analysis was conducted employing the inverse variance-weighted (IVW) MR to elucidate the potential causal link between peripheral metabolites and pancreatic cancer (Burgess et al., 2013). Additional methods were applied to assessed potential violations of MR assumptions, including maximum likelihood (Burgess et al., 2016), weighted median (Bowden et al., 2016), and MR-Egger (Bowden et al., 2015). The primary method employed was the IVW approach, which assumed the validity of all SNPs used as instrumental variables. In cases where a minimum of 50% of the weight was derived from valid instrument variants, the weighted median (WM) method was employed to ensure consistent estimations. For the evaluation of causal effects while accounting for potential horizontal pleiotropy, the MR-Egger regression method was utilized, albeit with a potential trade-off in precision. Simultaneously, the simple mode method was also utilized, positing a direct causal link between genetic variants associated with the exposure and the outcome, without accommodating the intricacies or biases intrinsic to more intricate MR methodologies. Comprehensive explanations of these methodologies can be obtained from previous scholarly publications (Burgess et al., 2013; Burgess et al., 2016; Bowden et al., 2015; Bowden et al., 2016). To explore the causal relationship between peripheral metabolites and pancreatic cancer, the Steiger test was employed (Hemani et al., 2017).

To verify the robustness of our findings, several sensitivity analyses were executed. Initially, the Cochran’s Q test was applied to evaluate the presence of heterogeneity within the data (Bowden et al., 2019). Subsequently, MR-Pleiotropy Residual Sum and Outlier (MR-PRESSO) methodology were employed to detect and eliminate SNPs with potential horizontal pleiotropic outliers, thus mitigating the potential impact of pleiotropy on the causal estimations (Verbanck et al., 2018). In cases where significant horizontal pleiotropy was detected in the MR-PRESSO global test, SNPs identified as outliers (with a significance level of *p* < 0.05) were excluded, and the remaining SNPs underwent re-analysis. Furthermore, the MR-Egger regression intercept was employed to assess potential pleiotropy in the SNPs, with a *p*-value ≥0.05 indicating the absence of horizontal pleiotropy. Additionally, we conducted a two-sample MR analysis on each individual SNP. Lastly, a leave-one-out analysis was carried out to detect any pleiotropy stemming from each individual SNP. All these analytical procedures were performed utilizing the TwoSampleMR packages. The outcomes were presented in terms of odds ratios (OR) alongside their corresponding 95% confidence intervals (CI). All *p*-values reported were two-sided, and statistical significance was defined at the 5% threshold.

## 3 Results

### 3.1 Causal effects of the peripheral metabolites on PC

The GWAS data of peripheral metabolites was obtained from three previously published study, which contained 7824, 24925, and 115078 samples of European ancestry, respectively. Supplementary Table S1 contains comprehensive information on the peripheral-metabolite-associated SNPs, including beta values, standard errors, effect alleles, other alleles, *etc.* The F-statistic values of selected SNPs ranged from 22.81 to 4176.17, supporting the absence of weak instrument bias.

**FIGURE 2:**
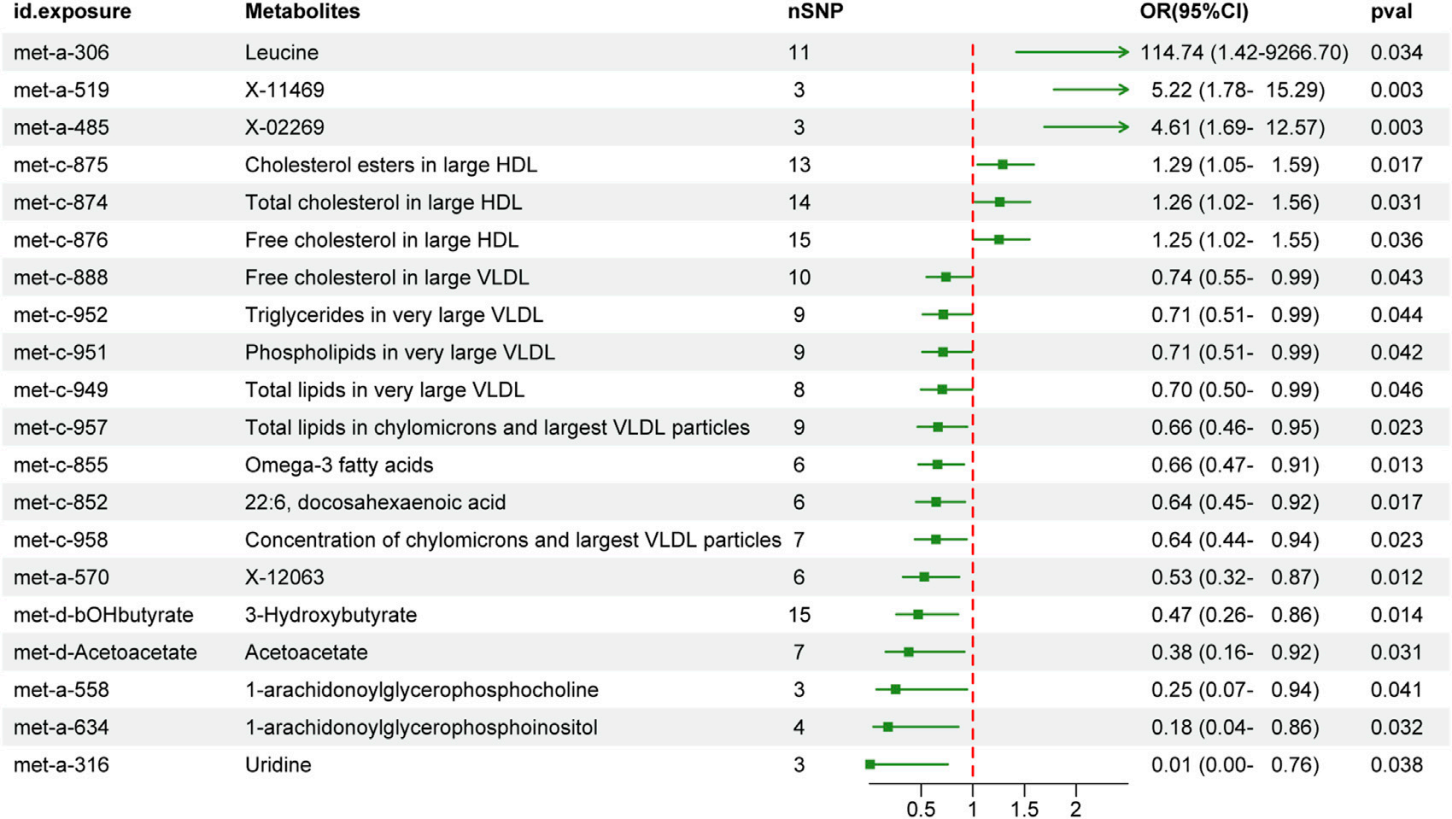
The effect of genetically determined peripheral metabolites on the risk of pancreatic cancer in the IVW results. OR, odds ratios; CI, confidence interval; IVW, inverse variance-weighted.

We first performed MR analysis to investigate the causal effects of peripheral metabolites on PC. The IVW-MR results demonstrated 5 and 15 metabolites which were positively and negatively causal associated with PC, respectively (Figure 2). Leucine (OR 114.74, 95% CI 1.42–9266.7, *p* = 0.034), X-11469 (OR 5.22, 95% CI 1.78–15.29, *p* = 0.003) and X-02269 (OR 4.61, 95% CI 1.69–12.57, *p* = 0.003) were the top three metabolites indicating an increasing PC risk. Remarkably, the HDL and VLDL particles occupied 45% of all significant 20 metabolites. Specifically, cholesterol esters in large HDL (OR 1.29, 95% CI 1.05–1.59, *p* = 0.017), total cholesterol in large HDL (OR 1.26, 95% CI 1.02–1.56, *p* = 0.031) and free cholesterol in large HDL (OR 1.25, 95% CI 1.02–1.55, *p* = 0.036) were associated with an elevated risk of PC whilst free cholesterol in large VLDL (OR 0.74, 95% CI 0.55–0.99, *p* = 0.043), triglycerides in very large VLDL (OR 0.71, 95% CI 0.51–0.99, *p* = 0.044), phospholipids in very large VLDL (OR 0.71, 95% CI 0.51–0.99, *p* = 0.042), total lipids in very large VLDL (OR 0.7, 95% CI 0.5–0.99, *p* = 0.046), total lipids in chylomicrons and largest VLDL particles (OR 0.66, 95% CI 0.46–0.95, *p* = 0.023) and concentration of chylomicrons and largest VLDL particles (OR 0.64, 95% CI 0.44–0.94, *p* = 0.023) demonstrated the opposite trend. In addition, omega-3 fatty acids (OR 0.66, 95% CI 0.47–0.91, *p* = 0.013), docosahexaenoic acid (22:6) (OR 0.64, 95% CI 0.45–0.92, *p* = 0.017), X-12063 (OR 0.53, 95% CI 0.32–0.87, *p* = 0.012), 3-Hydroxybutyrate (OR 0.47, 95% CI 0.26–0.86, *p* = 0.014), acetoacetate (OR 0.38, 95% CI 0.16–0.92, *p* = 0.031), 1-arachidonoylglycerophosphocholine (OR 0.25, 95% CI 0.07–0.94, *p* = 0.041), 1-arachidonoylglycerophosphoinositol (OR 0.18, 95% CI 0.04–0.86, *p* = 0.032) and uridine (OR 0.01, 95% CI 0–0.76, *p* = 0.038) were related to reduced PC risk.

Next, potential violations of MR assumptions were assessed by performing additional MR analyses using three alternative methods: maximum likelihood, weighted median and MR Egger (Supplementary Table S2). The overall trends of the effects of peripheral metabolites on PC analyzed by the additional methods mentioned above remained consistent, although some of them did not reach statistical significance (Supplementary Tables S2, S3). In contrast, MR-Egger regression yielded that leucine (OR 1.45e-05, 95% CI 1.73e-17-1.22e+7), X-02269 (OR 0.19, 95% CI 6.30e-5-564.38) and uridine (OR 1.41, 95% CI 1.20e-9-1.65e+11) was in the opposite direction of the IVW estimation, but it also did not reach statistical significance.

To evaluate heterogeneity and identify potential outliers, we conducted Cochran’s Q test and MR-PRESSO test, respectively. The results (Supplementary Table S3) indicated no significant heterogeneity (PQ > 0.05) and no outliers were detected. Additionally, all *p*-values from the MR-Egger intercept test were greater than 0.05, suggesting the absence of horizontal pleiotropy. Steiger filtering revealed that all genetic instrumental variables used for PC explained more variance in peripheral metabolites than in PC (Supplementary Table S2). To further assess the robustness of our findings, we employed several additional analyses. Firstly, we visually examined the forest plot (Supplementary Figure S1). Secondly, we utilized four methods to assess the results of the MR analysis, and a scatter plot specifically for PC was generated (Supplementary Figure S2). Finally, leave-one-out analysis further confirmed the robustness of our main results (Supplementary Figure S3).

### 3.2 Causal effects of the PC on peripheral metabolites

We incorporated a total of 11 independent SNPs as IVs for PC from the FinnGen database (Infantino et al., 2021). The detailed information of selected SNP was presented in Supplementary Table S4. No bias of weak instrument was confirmed by F statistics, which ranged from 20.87 to 32.06.

**FIGURE 3:**
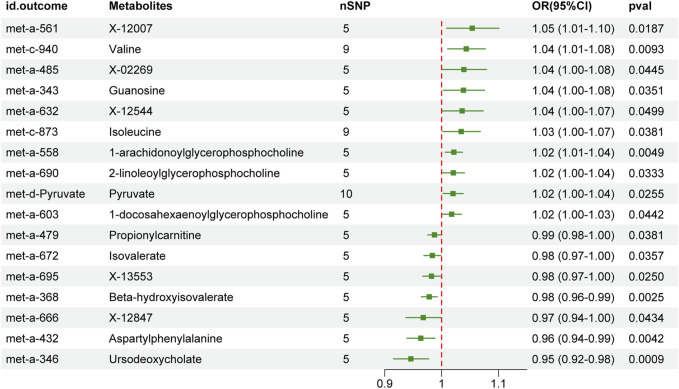
The effect of genetically determined pancreatic cancer on the alternation of peripheral metabolites in the IVW results. OR, odds ratios; CI, confidence interval; IVW, inverse variance-weighted.

The IVW-MR analysis demonstrated a significant causal relationship of PC with 17 peripheral metabolites (Figure 3). Specifically, PC was associated with increasing of X-12007 (OR 1.05, 95% CI 1.01–1.1, *p* = 0.019), valine (OR 1.04, 95% CI 1.01–1.08, *p* = 0.009), X-02269 (OR 1.04, 95% CI 1–1.08, *p* = 0.044), guanosine (OR 1.04, 95% CI 1–1.08, *p* = 0.035), X-12544 (OR 1.04, 95% CI 1–1.07, *p* < 0.05), isoleucine (OR 1.03, 95% CI 1–1.07, *p* = 0.038), 1-arachidonoylglycerophosphocholine (OR 1.02, 95% CI 1.01–1.04, *p* = 0.005), 2-linoleoylglycerophosphocholine (OR 1.02, 95% CI 1–1.04, *p* = 0.033), pyruvate (OR 1.02, 95% CI 1–1.04, *p* = 0.026) and 1-docosahexaenoylglycerophosphocholine (OR 1.02, 95% CI 1–1.03, *p* = 0.044); while decreasing of propionylcarnitine (OR 0.99, 95% CI 0.98–1, *p* = 0.038), isovalerate (OR 0.98, 95% CI 0.97–1, *p* = 0.036), X-13553 (OR 0.98, 95% CI 0.97–1, *p* = 0.025), beta-hydroxyisovalerate (OR 0.98, 95% CI 0.96–0.99, *p* = 0.003), X-12847 (OR 0.97, 95% CI 0.94–1, *p* = 0.043), aspartylphenylalanine (OR 0.96, 95% CI 0.94–0.99, *p* = 0.004) and ursodeoxycholate (OR 0.95, 95% CI 0.92–0.98, *p* = 0.001) (Figure 3A).

The additional analyses using the maximum likelihood, weighted-median and MR Egger methods consistently yielded a comparable consistent trend of PC on peripheral metabolites to the IVW method (Supplementary Table S5). The MR-Egger results demonstrated that PC-related metabolites, including X-02269 (OR 0.39), guanosine (OR 0.83), X-12544 (OR 0.48), 2-linoleoylglycerophosphocholine (OR 0.57), 1-docosahexaenoylglycerophosphocholine (OR 0.99), propionylcarnitine (OR 1.58), X-13553 (OR 1.05), X-12847 (OR 1.27), aspartylphenylalanine (OR 3.08) and ursodeoxycholate (OR 3.98), were contrast to IVW results although neither of them reached significant. The MR-Egger intercept test suggested no evidence of pleiotropy and MR-PRESSO global test indicated the absence of horizontal pleiotropic (Supplementary Table S6). No significant heterogeneity was observed based on Cochran’s Q statistics when considering non-outlier single IVs within the PC cohort. Scatter plots present the individual SNP effect and combined effect from each method for each outcome dataset (Supplementary Figure S4). The leave-one-out analyses confirmed the stability of the results even after removing one SNP at a time (Supplementary Figure S5). Steiger filtering indicated that the selected genetic IVs for PC exhibited a higher degree of variance explanation in PC compared to peripheral metabolites (Supplementary Table S5).

**FIGURE 4:**
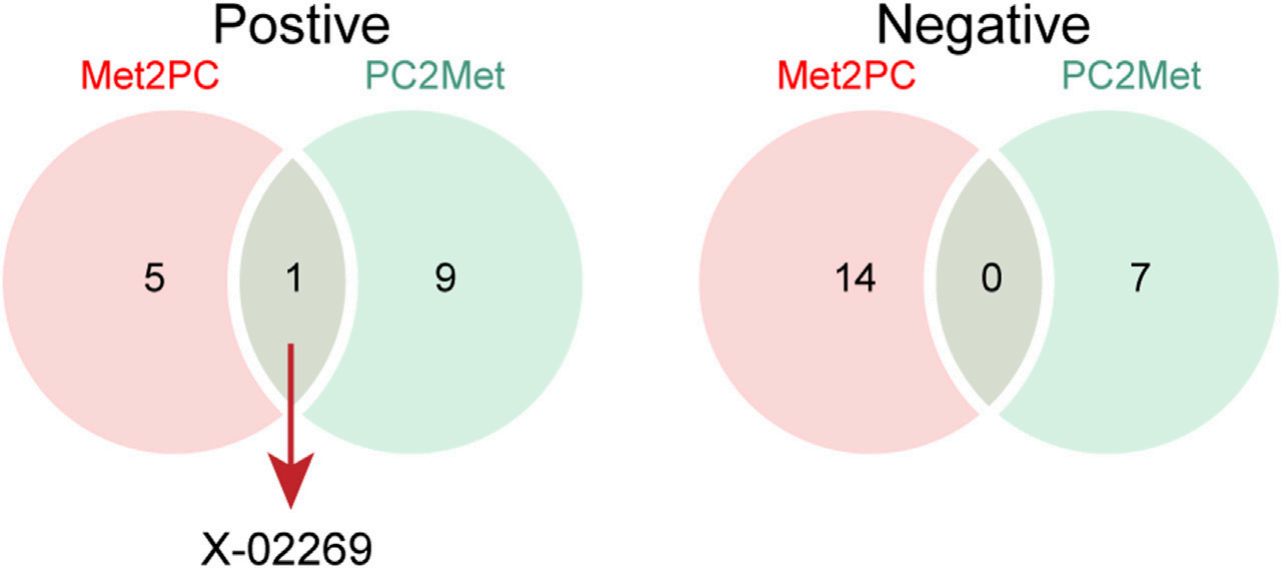
The Venn plot of positive (left) and negative (right) bidirectional two-sample Mendelian randomization IVW results. Met2PC represents the effect of peripheral metabolites on pancreatic cancer; PC2Met represents the effect of pancreatic cancer on peripheral metabolites.

We next compared the bi-causal relationships of PC and peripheral metabolites to find out the existence of positive feedback in PC development. Interestingly, X-02269 and PC formed a positive causal relationship pair (Figure 4), highlighting that X-02269 and PC might form a vicious circle in PC progression. We also found that 1-arachidonoylglycerophosphocholine indicated a lower risk of PC while PC demonstrated a higher level of it (Figure 2 and Figure 3). The result implied that 1-arachidonoylglycerophosphocholine might serve as a negative feedback node in PC development.

## 4 Discussion

PC stands as an ongoing challenge to global health due to its aggressive behavior and bleak prognosis (Qin et al., 2020; Casolino et al., 2021). The systemic metabolic status might play a crucial role in both PC development and non-neoplastic organ function. Despite advancements in cancer research, the intricate interplay between PC and peripheral metabolites remains largely unexplored. To illuminate the causal relationships between PC and peripheral metabolite profiles, we applied a comprehensive bidirectional tow-sample MR analysis. In our investigation, we discerned a spectrum of 20 peripheral metabolites intricately linked with the risk of PC. Furthermore, our study unveiled dynamic alterations in the levels of 17 metabolites in response to PC, thereby offering promising avenues for both early diagnostic strategies and therapeutic interventions. Notably, leucine, exhibiting the highest OR for PC causation in our analysis, has been previously reported to stimulate the growth of pancreatic cancer (Otsuru et al., 2019). Conversely, studies have reported that the restriction of leucine intake can suppress PC cells growth through the induction of apoptosis (Kamphorst et al., 2015). The detailed mechanisms underlying leucine’s promotion effect on PC may involve the activation of mTOR signaling and the promotion of tumor-related inflammation (Liu et al., 2014; Otsuru et al., 2019).

Interestingly, our findings underscore the role of lipid metabolic dysregulation as a significant regulator in PC progression. HDL particles exhibited an augmented PC risk, while VLDL particles demonstrated the converse trend. Cholesterol and cholesterol esters (CEs) within large HDL emerged as indicators of heightened PC risk in our analysis. This observation is corroborated by studies suggesting that HDL-mediated cholesterol depletion from PC cells restrains tumor development both *in vivo* and *in vitro* (Oberle et al., 2022). Moreover, the overexpression of SR-B1, a physiologically pertinent HDL receptor, in numerous tumors, including PC, has been implicated in promoting tumor growth (Rajora and Zheng, 2016; Oberle et al., 2022). This occurred through its binding to lipoproteins on the surface of HDL and facilitating lipid delivery, as well as the uptake of HDL-CE into the cell via a pore formed by SR-B1 (Valacchi et al., 2011). In contrast, the causal relationship of VLDL particles with PC was reversed compared to HDL particles in our results. VLDL, originating in the liver, facilitates the transfer of triglycerides from the liver to peripheral tissues. During its transit in the bloodstream, triglycerides are progressively removed, leading to the conversion of VLDL to intermediate-density lipoprotein (IDL) and subsequently to LDL. Despite this, scant research has focused on VLDL as opposed to LDL in PC. Some studies have suggested that genetically elevated LDL-cholesterol levels are associated with pancreatic cancer, and inhibiting LDLR in PC cells considerably reduces cholesterol uptake and alters its distribution, leading to decreased cancer cell proliferation (Jung et al., 2021; Yin et al., 2022). These observations may underscore the potential pathological significance of the transformation from VLDL to LDL in PC development. In conclusion, these results highlighted the pronounced heterogeneity of the HDL and VLDL particle pool and their interaction with PC cells or non-malignant cells in the context of disease progression, warranting further in-depth exploration.

The interplay between PC and peripheral metabolites was further substantiated through reverse MR analysis. Seventeen metabolites displayed altered levels in response to PC. Notably, two BCAAs, valine and isoleucine, displayed heightened levels under PC influence, contrary to prior studies reporting BCAA inhibition in PC, even though serum BCAA levels were found to be elevated in PC rats compared to those with chronic pancreatitis (Fang et al., 2007; Li et al., 2021). Moreover, enhanced BCAA catabolism by BCAT2 plays a pivotal role in the development of PC, and this role is further exacerbated by KRAS mutations (Li et al., 2020). Another significant metabolite type elevated by PC was derivatives of glycerophospholipids, including 1-arachidonoylglycerophosphocholine, 2-linoleoylglycerophosphocholine, and 1-docosahexaenoylglycerophosphocholine. Targeting glycerophospholipid synthesis has been suggested as a potential strategy to enhance cytotoxic drug sensitivity (Kaoutari et al., 2021). Furthermore, HIF-1 has the capacity to promote lipid droplet accumulation and enhance cancer cell viability under hypoxic conditions by directly targeting AGPAT2, an enzyme crucially involved in the glycerophospholipid/triacylglycerol biosynthesis pathway (Triantafyllou et al., 2018). The notable activity of pyruvate kinase type M2, culminating in pyruvate synthesis in PC, aligns with our findings of increased peripheral pyruvate levels (Cameron et al., 2018).

By cross-referencing the results of the bidirectional two-sample MR analysis, we identified two shared metabolites—X-02269 and 1-arachidonoylglycerophosphocholine. The mutual causation observed between X-02269 and PC implies their potential involvement in a vicious circle in PC onset and progression. Unfortunately, X-02269 remains inadequately investigated as a novel metabolite. Additionally, 1-arachidonoylglycerophosphocholine indicated a reduced PC risk and an increase under PC influence, possibly serving as a negative feedback regulator. However, there is a lack of research on 1-arachidonoylglycerophosphocholine and its relationship with PC, as far as we know, indicating the need for further studies in this area.

Despite the comprehensive nature of our MR analysis in elucidating the causal links between peripheral metabolites and PC, several limitations warrant consideration. Firstly, our conclusions are solely derived from MR analysis, necessitating experimental validation. Secondly, while we focused on the relationship between PC and peripheral metabolites, the role of local metabolites within the PC tissue remains unexplored. Thirdly, although we established causal connections between PC and metabolites, the underlying mechanisms necessitate further investigation, particularly regarding specific cell types and signaling pathways. Lastly, our study’s confinement to a single cancer type and dataset prompts us to acknowledge the potential for intriguing revelations through systematic analyses across diverse cancer types and cohorts.

## 5 Conclusion

The bidirectional MR analysis uncovers the intricate interplay between peripheral metabolites and PC, offering novel insights into PC etiology. Our findings contribute to the broader understanding of PC biology and may guide the development of innovative strategies for early diagnosis and intervention. Further research is warranted to elucidate the precise molecular mechanisms underlying the observed causal relationships and to explore the clinical implications of the identified metabolites.

## Data Availability

The original contributions presented in the study are included in the article/Supplementary Material, further inquiries can be directed to the corresponding authors.
