# Supplementary figures and images for: Unveiling the intricate causal nexus between pancreatic cancer and peripheral metabolites through a comprehensive bidirectional two-sample Mendelian randomization analysis
Source: Front Mol Biosci. 2023 Oct 25;10:1279157. doi: 10.3389/fmolb.2023.1279157 (PMC10634252; doi:10.3389/fmolb.2023.1279157)

# MR Test

Inverse variance weighted

Maximum likelihood

MR Egger

Weighted median

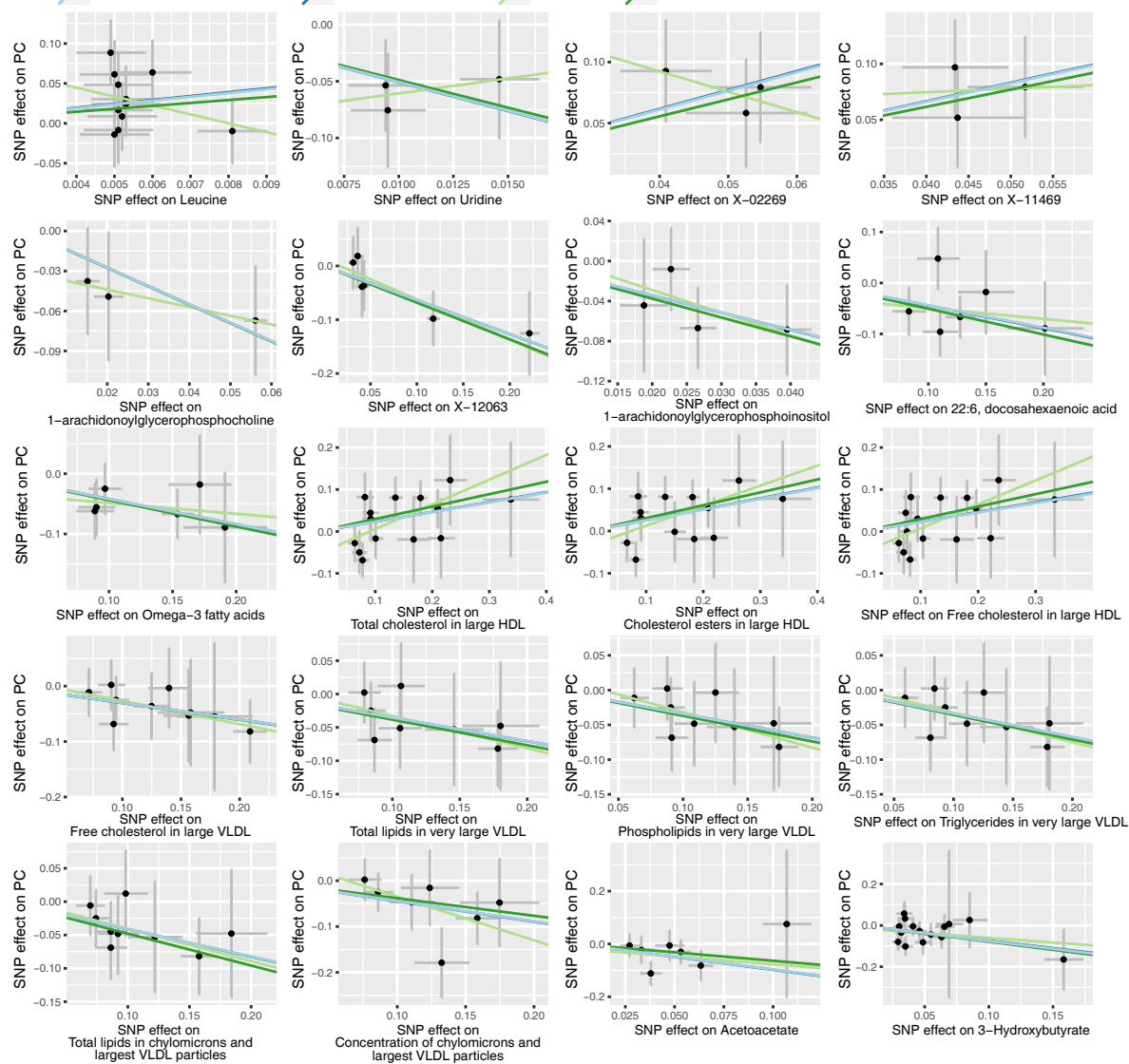

Supplement: Supplementary file 3 [file Image5.PDF]

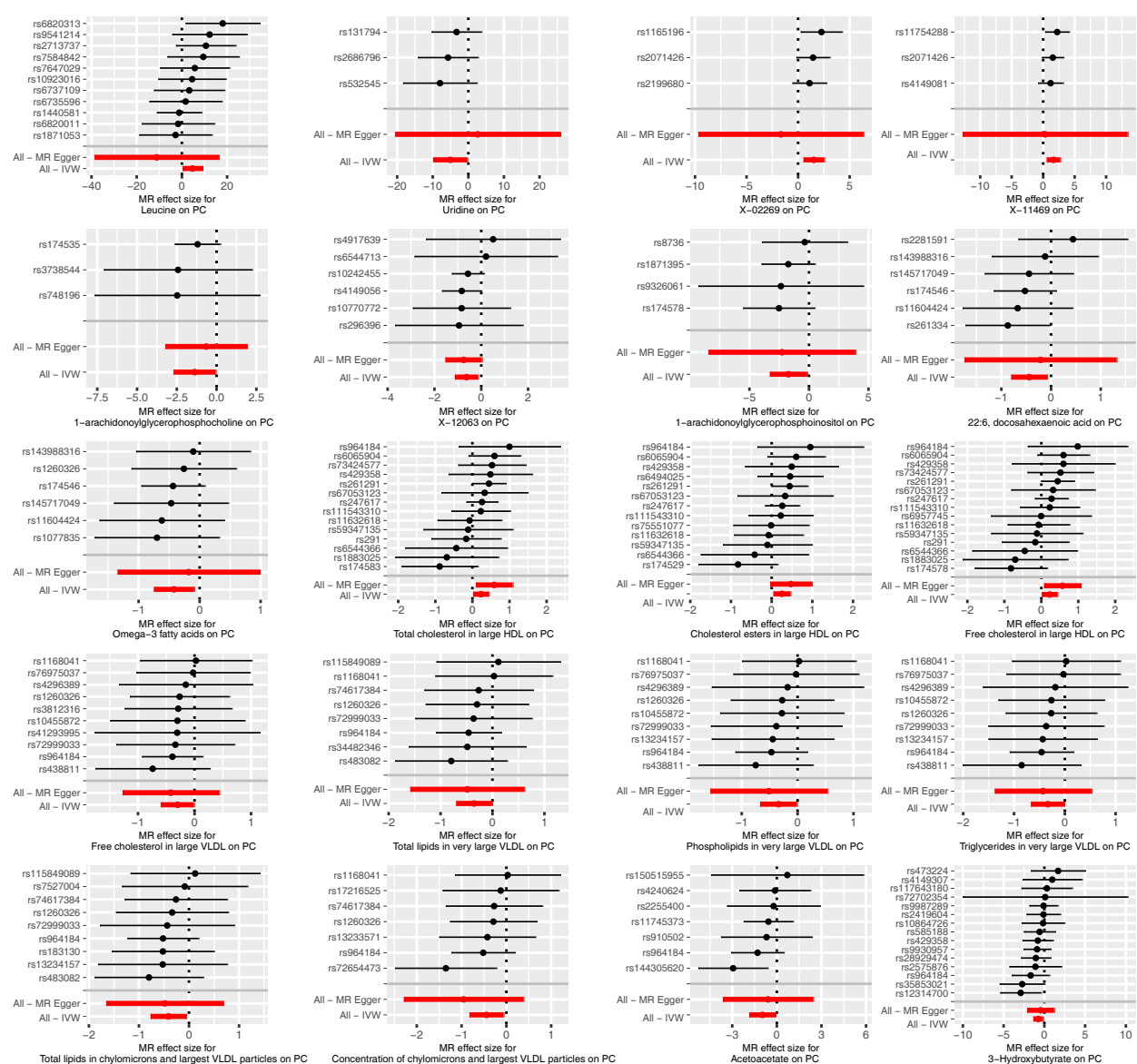

Supplement: Supplementary file 4 [file Image4.PDF]

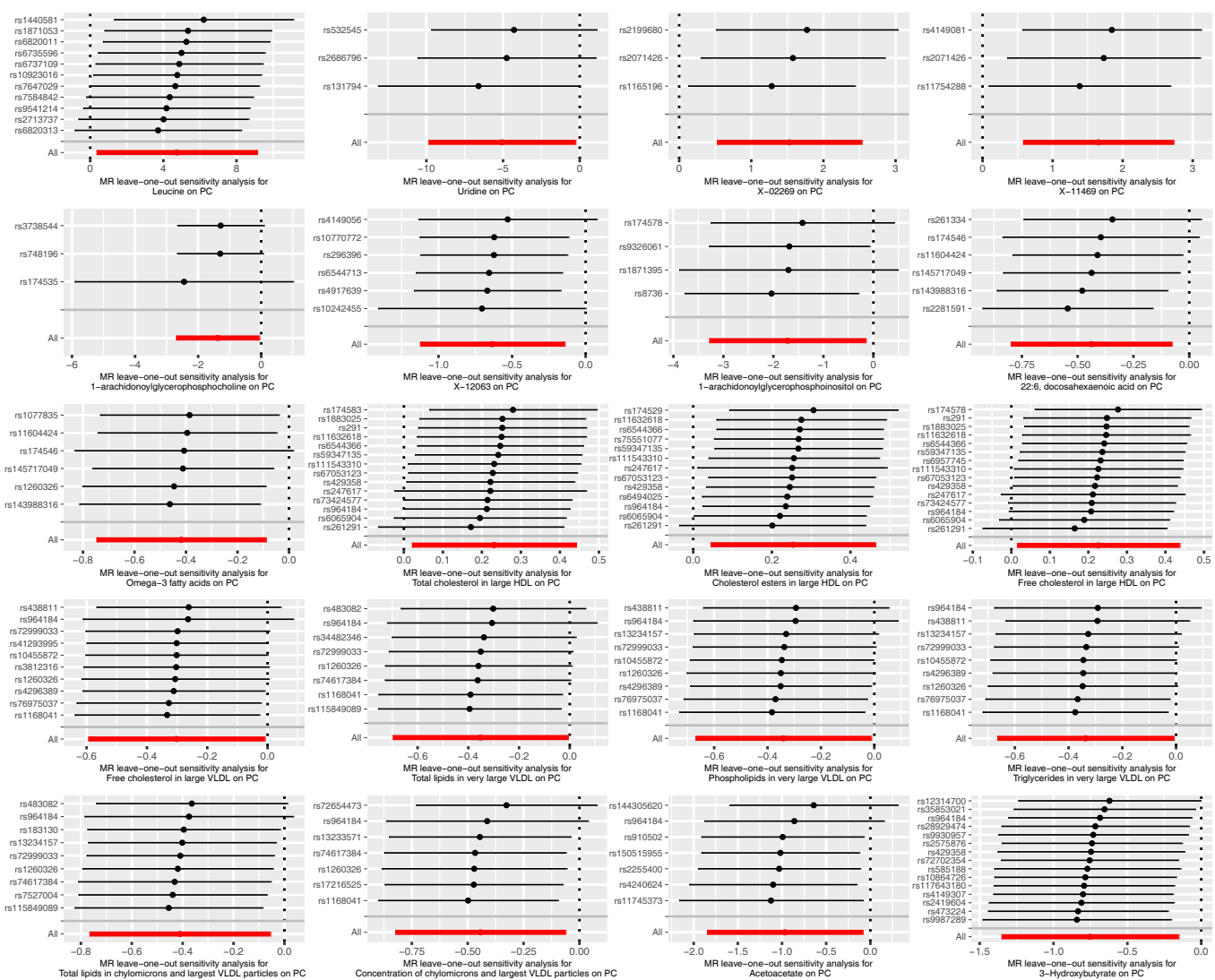

Supplement: Supplementary file 5 [file Image2.PDF]

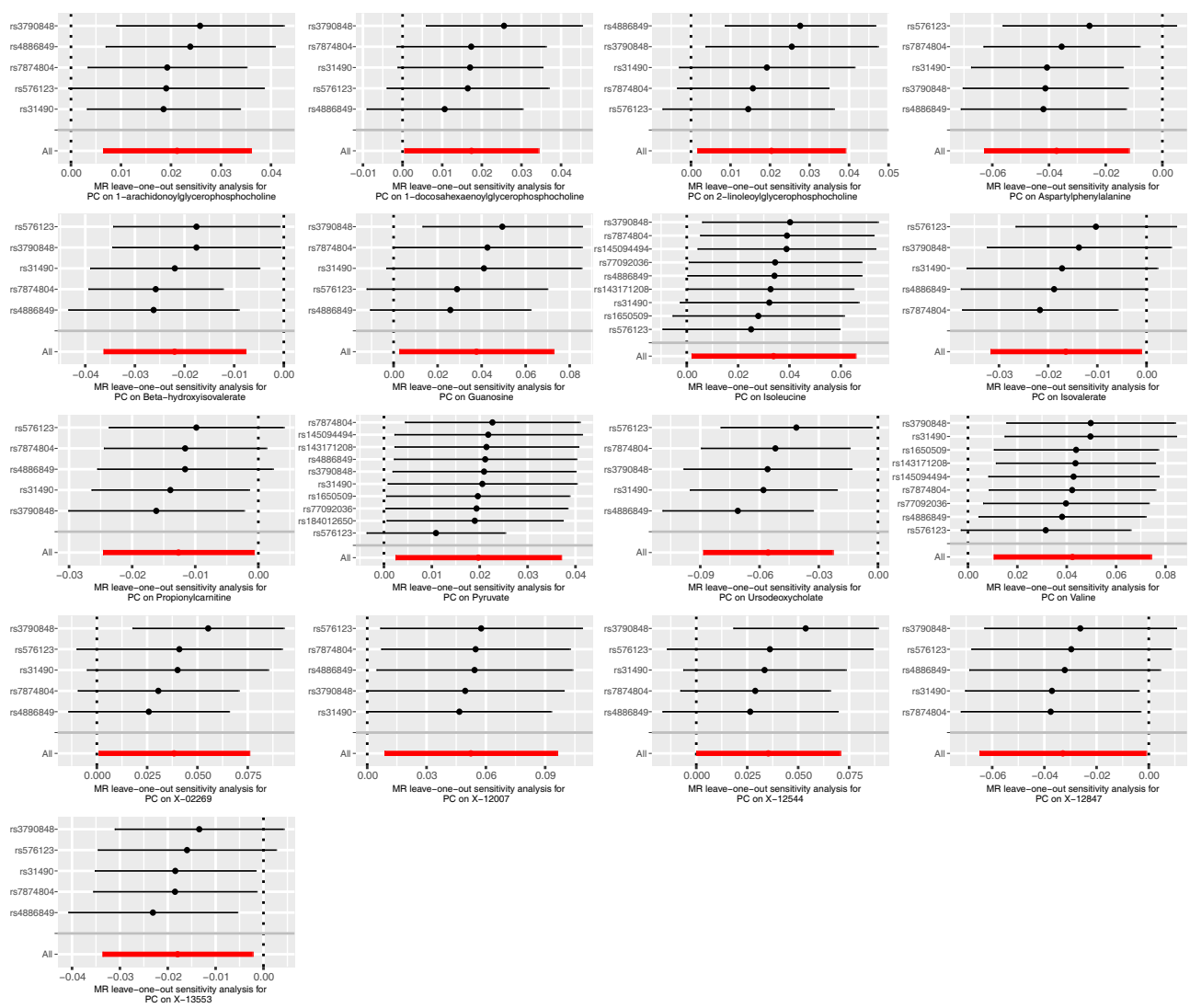

Supplement: Supplementary file 6 [file Image3.PDF]

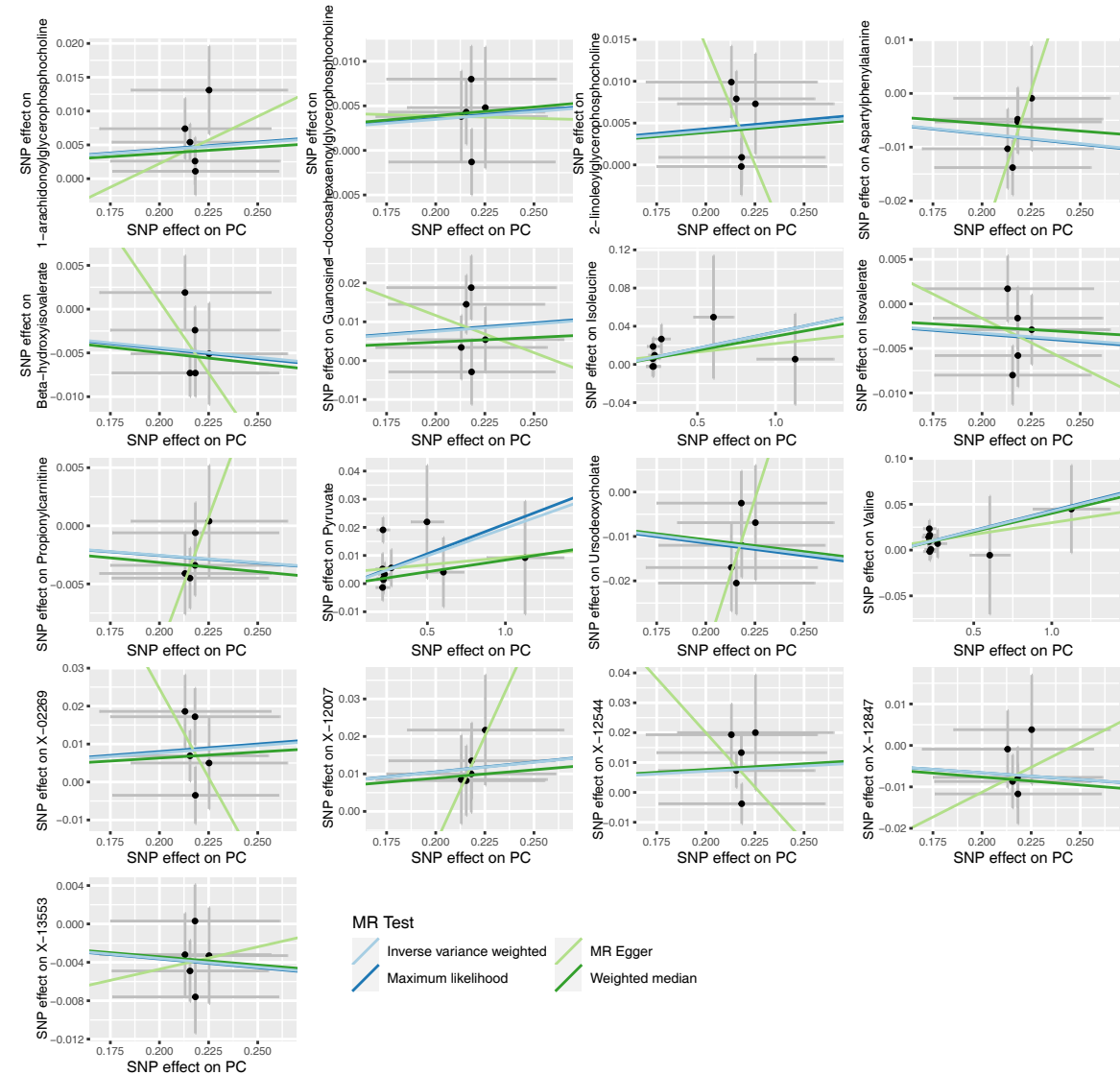

Supplement: Supplementary file 11 [file Image1.PDF]
